# Comparison Between Structural and Compositional Changes of Human Root Surface on Exposure to Different Wavelengths of Diode Lasers for Different Time Frames: An In-Vitro Study

**DOI:** 10.7759/cureus.108933

**Published:** 2026-05-15

**Authors:** Abhinandan K Bokriya, Pramod F Waghmare, Prashant Rao, Devashri Newaskar, Manasi Yewale, Sanpreet S Sachdev

**Affiliations:** 1 Periodontology, Bharati Vidyapeeth (Deemed to be University) Dental College and Hospital, Pune, IND; 2 Oral Pathology and Microbiology, Bharati Vidyapeeth (Deemed to be University) Dental College and Hospital, Pune, IND; 3 Oral Pathology and Microbiology, Bharati Vidyapeeth (Deemed to be University) Dental College and Hospital, Navi Mumbai, IND

**Keywords:** diode laser, energy-dispersive x-ray analysis, periodontitis, root surface roughness, scanning electron microscopy

## Abstract

Background

Diode lasers are increasingly used as adjuncts in periodontal therapy because of their potential for root surface decontamination and biofilm reduction. However, their effects on the structural and compositional integrity of the root surface depend on the irradiation parameters used.

Aim

To evaluate and compare the structural and compositional changes in the roots of extracted human single-rooted permanent teeth after diode laser irradiation at 660 nm and 810 nm for 30 and 45 seconds.

Materials and methods

This in vitro study included 52 extracted human single-rooted permanent teeth, which were divided into four groups (n=13 each): Group 1, 660 nm for 30 seconds; Group 2, 660 nm for 45 seconds; Group 3, 810 nm for 30 seconds; and Group 4, 810 nm for 45 seconds. After laser application, surface roughness was assessed using Ra, Rq, and Rz parameters. Morphological changes were evaluated by scanning electron microscopy (SEM), and elemental composition was analyzed using energy-dispersive X-ray analysis (EDAX). Intergroup comparison of surface roughness was performed using Welch analysis of variance (ANOVA) followed by pairwise post hoc testing.

Results

Surface roughness increased progressively from Group 1 to Group 4. Mean Ra values ranged from 0.0031±0.0002 µm in Group 1 to 0.0123±0.0014 µm in Group 4. Similar trends were seen for Rq and Rz. Intergroup differences were statistically significant for Ra, Rq, and Rz (p<0.001 for all). Pairwise analysis showed no significant difference between Groups 1 and 2, whereas all other comparisons were significant. EDAX analysis showed increasing carbon and oxygen and decreasing phosphorus and calcium from Group 1 to Group 4. SEM findings also showed progressively greater surface alteration with increasing wavelength and exposure duration.

Conclusion

Diode laser irradiation produced wavelength- and time-dependent structural and compositional changes on the root surface. The greatest alterations were observed with 810 nm irradiation for 45 seconds, indicating that laser parameters should be selected cautiously to minimize excessive root surface damage.

## Introduction

Periodontitis is a chronic multifactorial inflammatory disease associated with dysbiotic plaque biofilms and is characterized by progressive destruction of the tooth-supporting tissues, which may ultimately compromise function and tooth retention [1]. Nonsurgical periodontal therapy remains the primary approach for managing periodontitis, and root surface debridement (RSD) continues to be its fundamental component because it aims to remove subgingival biofilm and calculus and create a biologically compatible root surface [2]. However, RSD is technique-sensitive, may be less effective in deep pockets and anatomically complex areas, and mechanical instrumentation can produce a smear layer on the root surface that may interfere with favorable periodontal healing and regeneration [2,3].

In recent years, laser-assisted periodontal therapy has been investigated as an adjunct to conventional debridement. Among the available systems, diode lasers have gained considerable attention because they are compact, relatively economical, and show good affinity for pigmented tissues and inflamed soft tissues [4]. Their use in periodontics has been associated with bacterial reduction, pocket decontamination, soft tissue curettage, haemostasis, and possible biostimulatory effects that may support wound healing [4,5]. Systematic reviews have also suggested that adjunctive diode laser application, when used along with RSD, may provide additional clinical benefits in selected periodontal situations, particularly in relation to probing depth reduction and improvement in inflammatory parameters [4,5].

Despite these potential advantages, the effect of diode laser irradiation on the root surface itself remains an important concern. Successful periodontal healing depends not only on disinfection of the periodontal pocket but also on preservation of a root surface that is biologically compatible for fibroblast attachment and connective tissue healing [6]. Experimental studies have shown that diode laser exposure can alter root surface morphology and composition, producing smoothing, melting, cracks, charring, or mineral changes depending on the wavelength and exposure duration used [7,8]. Therefore, understanding the structural and compositional effects of different diode laser parameters on root surfaces is essential before recommending their wider periodontal use.

In this context, the present in-vitro study was undertaken to evaluate and compare the structural and compositional changes in the roots of extracted human single-rooted permanent teeth after diode laser irradiation at 660 nm and 810 nm for 30 and 45 seconds. The objective was to assess the influence of wavelength and exposure duration on root surface characteristics and to identify parameters that may be more suitable for periodontal application.

## Materials and methods

The present in vitro study was conducted in the institutional department of Periodontology. The study protocol was approved by the Institutional Ethical Committee before commencement (BVDU/IEC/R1/15/23-24, dated: 31/08/2023), and ethical standards were maintained throughout the study, including during specimen collection and handling.

Sample selection

Only single-rooted extracted human permanent teeth extracted for orthodontic reasons were included in the study. Single-rooted teeth were selected to minimize anatomical variability and to permit more standardized irradiation and surface analysis. Inclusion of multirooted teeth could introduce confounding due to differences in root morphology, furcation anatomy, and access to the treatment surface. Teeth were excluded if they were carious, deciduous, multirooted, or fractured. The sample size was determined using OpenEpi version 3 (Emory University, Atlanta, USA). Sample size estimation was based on calcium weight percentage data reported by Yaprak et al., who evaluated the effect of 810 nm diode laser application on root surface elemental composition using scanning electron microscopy-energy dispersive x-ray analysis (SEM-EDX) and reported mean calcium values of 10.17±3.96 in intact root surfaces and 25.24±3.96 in laser-applied root surfaces [9]. These were distributed equally into four experimental groups, with 13 specimens in each group.

Specimen preparation

**Figure 1:**
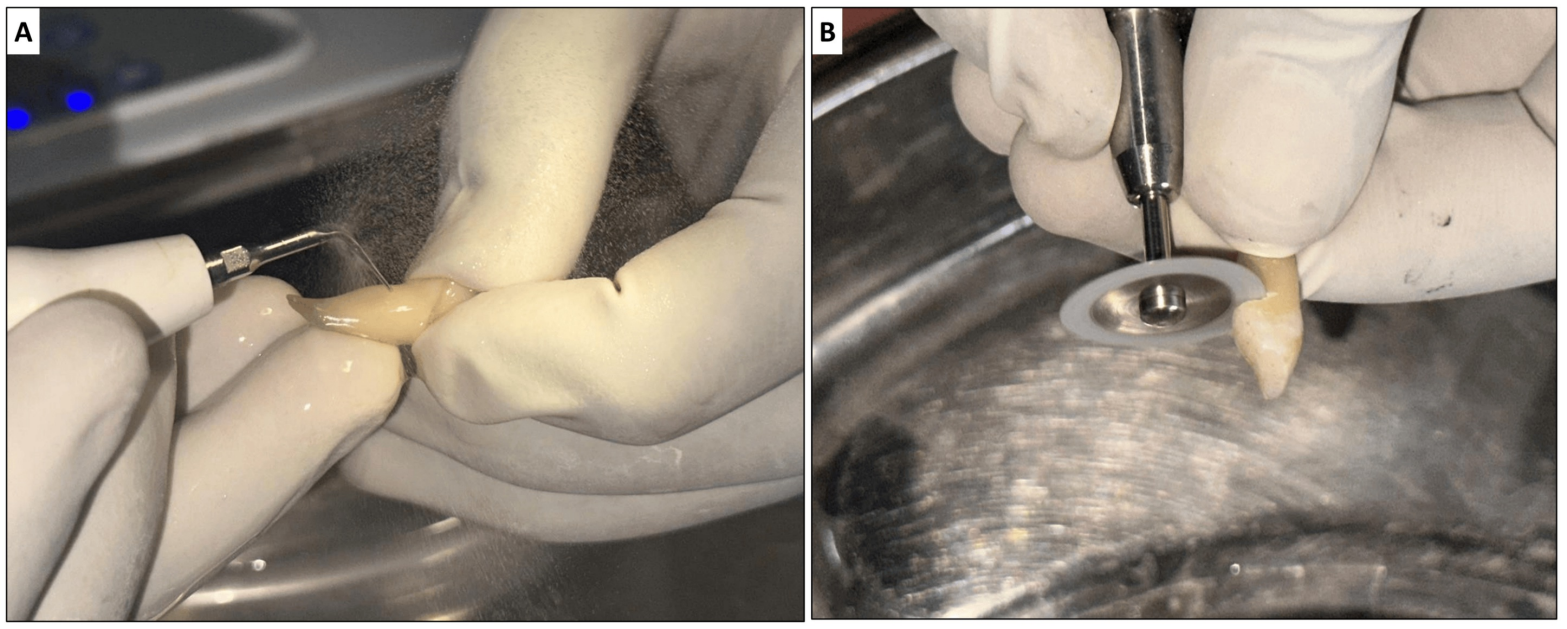
Preliminary preparation of the tooth specimen A) Root surface debridement of the specimen; B) Separation of crown and root of the tooth specimen using a diamond disc bur

After extraction under sterile conditions, RSD was performed for the selected teeth, which were then processed for laboratory evaluation. The crowns were separated from the roots using a low-speed diamond disc bur, taking care to avoid introducing additional damage to the root surface by safeguarding it with fingers (Figure 1). Each root specimen was then thoroughly cleaned to remove residual tissue and debris that could interfere with laser delivery or surface analysis.

Group allocation

The prepared root specimens were divided into four groups according to the wavelength of the diode laser and the duration of exposure. The randomization was performed by a simple randomization method using a computer-generated random sequence, and an allocation ratio of 1:1:1 was maintained. Group 1 received diode laser irradiation at 660 nm for 30 seconds. Group 2 received diode laser irradiation at 660 nm for 45 seconds. Group 3 received diode laser irradiation at 810 nm for 30 seconds. Group 4 received diode laser irradiation at 810 nm for 45 seconds.

**Figure 2:**
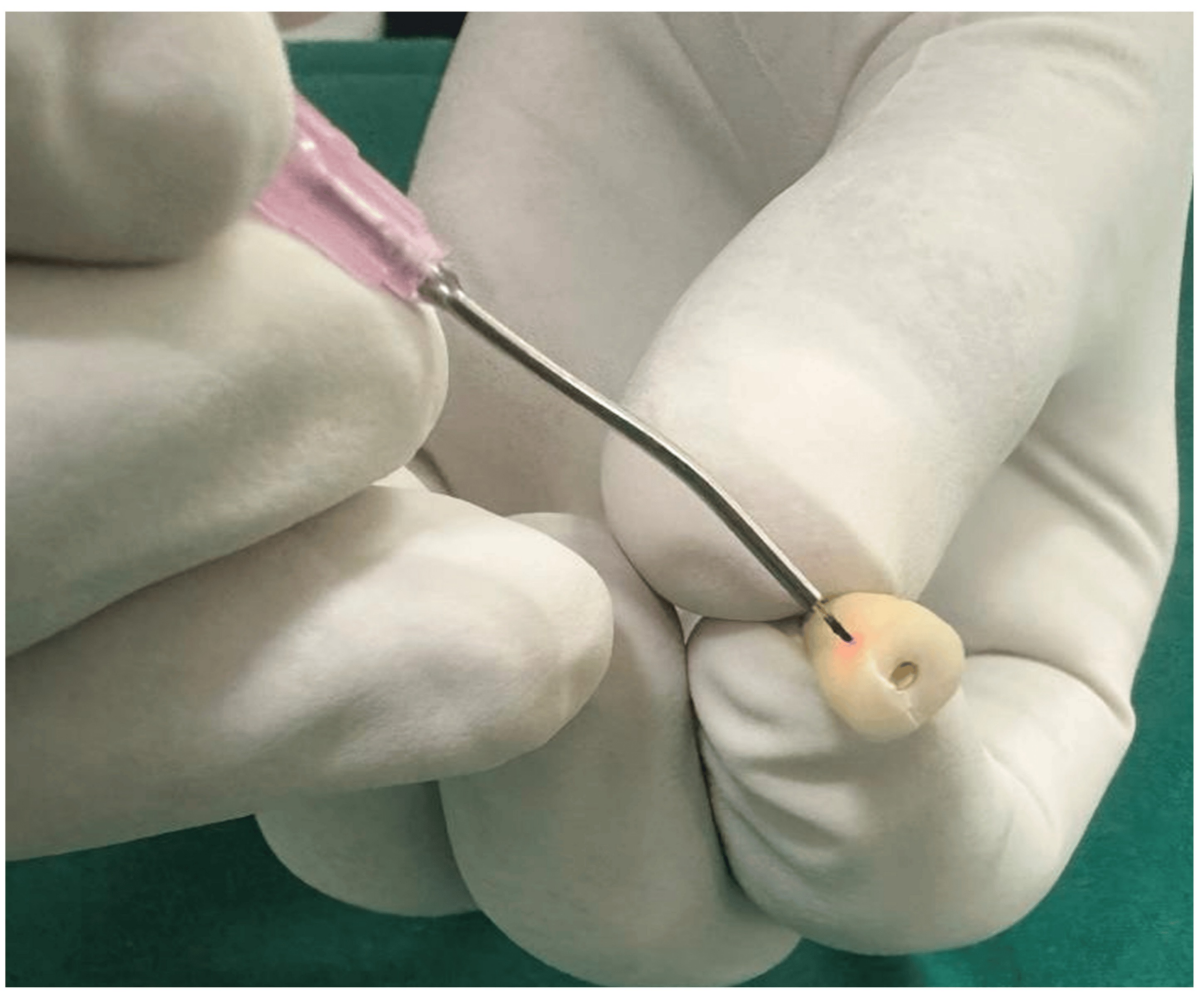
Irradiation of the tooth sample through diode laser tip

A diode laser system, Novolase Dual Wave Endo Diode (Novolase Technologies, India), capable of emitting both 660 nm and 810 nm wavelengths, was used for the experiment. During irradiation, the laser was applied directly to the root surface under controlled conditions. The treatment area on each root specimen was standardized and marked before irradiation. Laser application was performed over the marked area using a uniform scanning motion under standardized conditions for all specimens. The application was standardized by maintaining the laser tip at a distance of 1 mm from the root surface (Figure 2), and exposure conditions were kept uniform in order to minimize overheating and ensure consistency among specimens. The exposure protocol was standardized for all specimens according to group allocation: 660 nm for 30 seconds, 660 nm for 45 seconds, 810 nm for 30 seconds, and 810 nm for 45 seconds.

Outcome assessment

Following laser treatment, the specimens were subjected to post-treatment structural and compositional analysis. Structural assessment included evaluation of the treated root surface morphology and surface roughness. Micromorphological examination was performed using SEM, Zeiss Crossbeam 750 (Carl Zeiss Microscopy GmbH, Jena, Germany), with emphasis on surface irregularities, dentinal tubule exposure, dentin or cementum surface changes, and the presence or removal of the smear layer. In addition to qualitative surface examination, quantitative surface roughness analysis was performed using a surface roughness test (Mitutoyo SJ 210, Mitutoyo Corporation, Kawasaki, Japan) to record the parameters related to surface roughness. These comprised average roughness (Ra), roughness with more emphasis on larger irregularities (Rq), and peak-to-valley height (Rz) to compare the topographic changes among the four groups, and were recorded in micrometres (µm).

**Figure 3:**
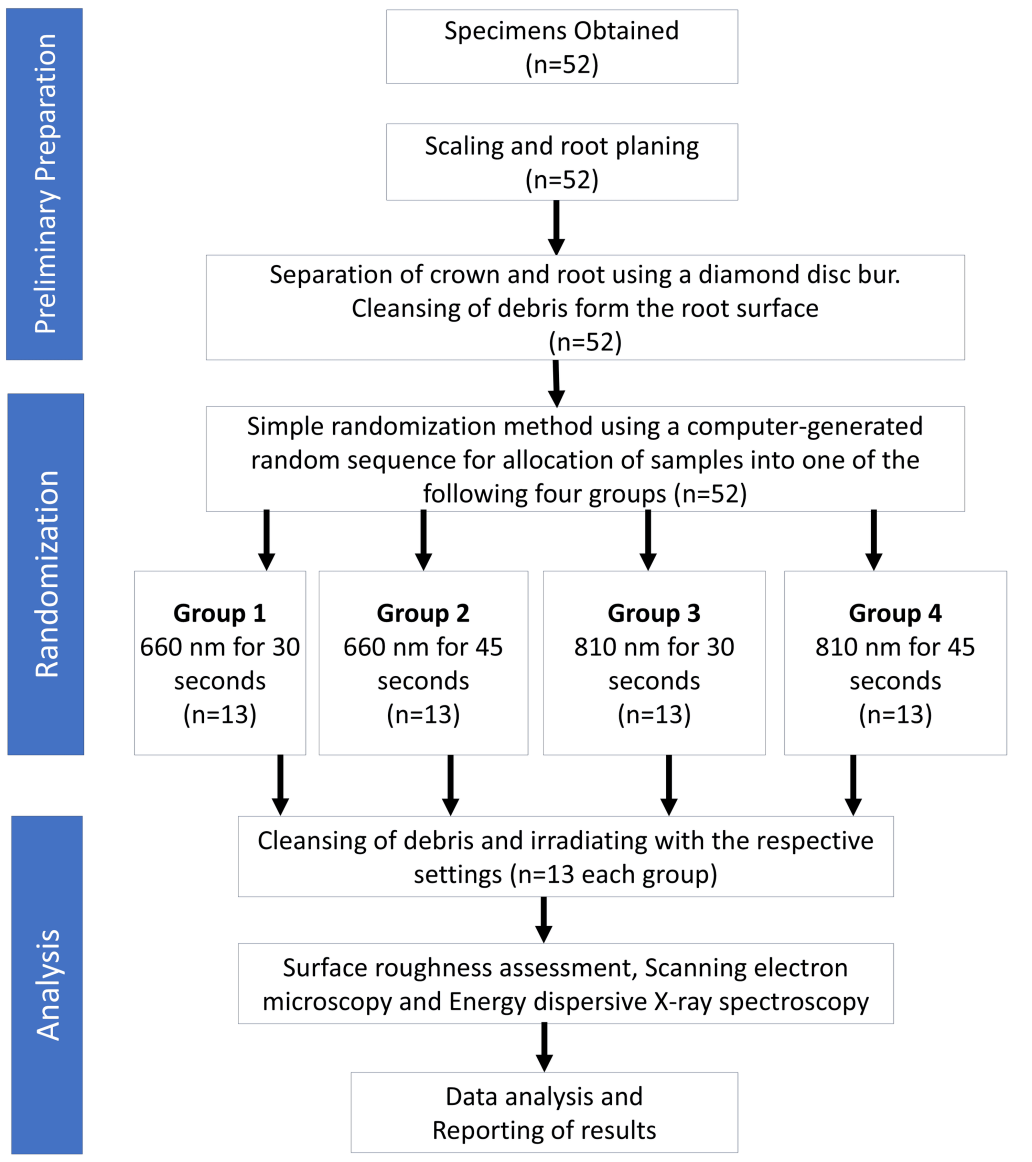
Flow diagram indicating the study procedures from obtaining and preparing specimens to data analysis

Compositional assessment was performed using EDAX (Iridium Ultra, South Africa) to determine the levels of carbon, oxygen, phosphorus, and calcium on the irradiated root surfaces. These elements were selected because they reflect the organic and inorganic composition of the root surface and therefore provide insight into mineral alteration and surface modification following laser exposure. The EDAX results were recorded group-wise to compare the relative compositional changes produced by the different irradiation protocols. The study processes are delineated in the form of a flow chart in Figure 3.

Statistical analysis

All study data were analyzed using SPSS software, version 25.0 (IBM Corporation, Armonk, USA). The software was used to compile and analyze the quantitative findings obtained from surface roughness assessment and EDAX evaluation across the four experimental groups. Group-wise comparison of the structural and compositional findings was then carried out on the basis of the recorded values for surface roughness parameters and elemental composition.

## Results

All 52 specimens were included in the analysis, with 13 specimens in each experimental group.

Surface roughness analysis

Surface roughness was evaluated using Ra, Rq, and Rz. Mean roughness values increased progressively from Group 1 to Group 4 for all three parameters. The lowest values were observed in Group 1 (660 nm, 30 s), whereas the highest values were recorded in Group 4 (810 nm, 45 s). Group 2 showed only a slight increase compared with Group 1, while Groups 3 and 4 demonstrated a marked increase in roughness. The descriptive statistics are presented in Table 1. Intergroup and pairwise post hoc comparisons are summarized in Table 2.

**Table 1:** Surface roughness parameters of root surfaces in the four study groups

Group	Laser setting	Ra (µm), mean±SD (range)	Rq (µm), mean±SD (range)	Rz (µm), mean±SD (range)
Group 1	660 nm, 30 s	0.0031±0.0002 (0.0028–0.0033)	0.0040±0.0002 (0.0035–0.0043)	0.0212±0.0017 (0.018–0.024)
Group 2	660 nm, 45 s	0.0033±0.0008 (0.0022–0.0045)	0.0041±0.0008 (0.0031–0.0052)	0.0227±0.0053 (0.015–0.030)
Group 3	810 nm, 30 s	0.0080±0.0011 (0.0065–0.0095)	0.0092±0.0011 (0.0077–0.0108)	0.0424±0.0051 (0.035–0.050)
Group 4	810 nm, 45 s	0.0123±0.0014 (0.0102–0.0145)	0.0141±0.0014 (0.0121–0.0162)	0.0640±0.0076 (0.053–0.075)

**Table 2:** Intergroup and pairwise post hoc comparison of surface roughness parameters p-value for overall intergroup comparison was obtained using Welch’s analysis of variance (ANOVA). Pairwise comparisons between groups were analyzed using the Welch t-test, with Holm correction applied for multiple comparisons. p<0.05=statistically significant.

Comparison	Ra test value	Ra p-value	Rq test value	Rq p-value	Rz test value	Rz p-value
Overall intergroup comparison (Welch ANOVA)	F(3,20.90)=255.78	<0.001	F(3,21.87)=287.43	<0.001	F(3,22.72)=178.36	<0.001
Group 1 vs Group 2	t(12.93)=-1.00	0.334	t(14.20)=-0.72	0.485	t(14.45)=-0.95	0.356
Group 1 vs Group 3	t(12.52)=-16.62	<0.001	t(13.09)=-16.78	<0.001	t(14.65)=-14.30	<0.001
Group 1 vs Group 4	t(12.29)=-23.50	<0.001	t(12.68)=-25.69	<0.001	t(13.20)=-19.90	<0.001
Group 2 vs Group 3	t(22.23)=-12.84	<0.001	t(21.51)=-13.62	<0.001	t(23.96)=-9.72	<0.001
Group 2 vs Group 4	t(18.84)=-20.14	<0.001	t(18.74)=-22.42	<0.001	t(21.42)=-16.16	<0.001
Group 3 vs Group 4	t(22.22)=-8.89	<0.001	t(22.78)=-9.80	<0.001	t(20.95)=-8.57	<0.001

Pairwise analysis showed no significant difference between Group 1 and Group 2 for Ra, Rq, or Rz. All other pairwise comparisons were statistically significant. Among the three roughness parameters, Rz showed the widest separation between the lowest- and highest-exposure groups, increasing from 0.0212±0.0017 µm in Group 1 to 0.0640±0.0076 µm in Group 4.

Elemental composition analysis

EDAX analysis showed a progressive compositional shift across the four groups. Carbon and oxygen increased from Group 1 to Group 4, whereas phosphorus and calcium decreased over the same sequence. The calculated Ca/P ratio also showed a gradual decline from Group 1 to Group 4. The elemental composition is presented in Table 3. From Group 1 to Group 4, carbon increased from 5.98% to 13.36% and oxygen from 17.65% to 39.54%, while phosphorus decreased from 15.47% to 9.25% and calcium from 35.65% to 16.85%. The greatest compositional alteration was observed in Group 4.

**Table 3:** Elemental composition of root surfaces across the four study groups

Parameter	Group 1	Group 2	Group 3	Group 4
Carbon (%)	5.98	7.20	11.65	13.36
Oxygen (%)	17.65	22.65	32.54	39.54
Phosphorus (%)	15.47	14.60	11.52	9.25
Calcium (%)	35.65	32.18	21.65	16.85
Ca/P ratio*	2.30	2.20	1.88	1.82

Scanning electron microscopic analysis

**Figure 4:**
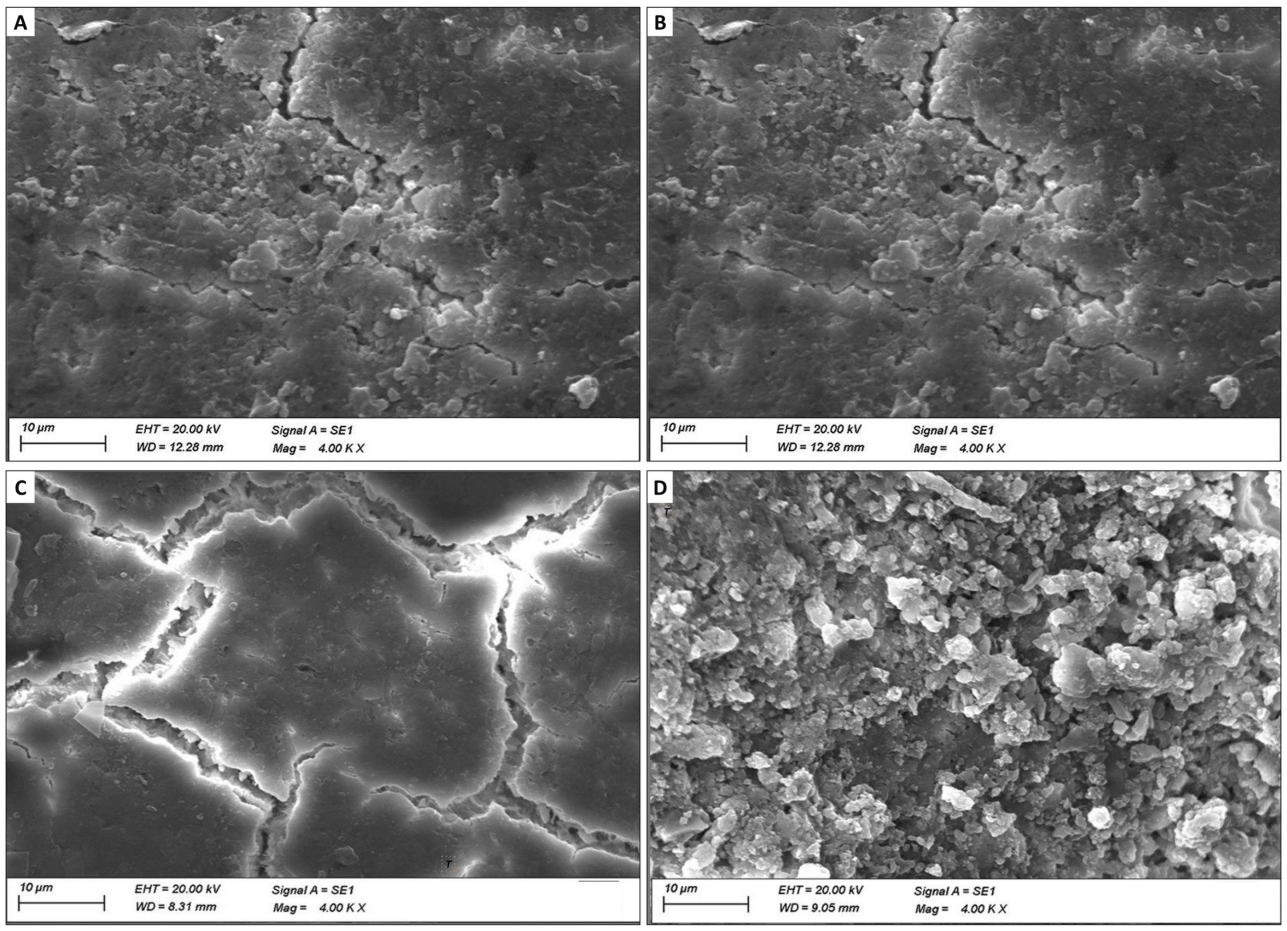
Representative scanning electron microscopy images of the four groups A) 660 nm for 30 seconds, B) 660 nm for 45 seconds, C) 810 nm for 30 seconds, and D) 810 nm for 45 seconds.

SEM examination showed progressive micromorphological alteration with increasing wavelength and exposure duration. Group 1 showed minimal surface alteration with light surface debris and negligible irregularities. Group 2 demonstrated more evident changes, including microcracks and greater debris accumulation. Group 3 showed further surface disruption with extensive cracking and irregular topography. Group 4 exhibited the most pronounced changes, characterized by charring, peeling of the cementum surface, and deep fissuring. Representative SEM images in Figure 4 illustrate a graded increase in surface damage from Group 1 through Group 4.

## Discussion

The present study showed a clear parameter-dependent effect of diode laser irradiation on the root surface. Surface roughness increased progressively from Group 1 to Group 4 for Ra, Rq, and Rz, and the EDAX profile showed a parallel increase in carbon and oxygen with a reduction in phosphorus, calcium, and Ca/P ratio. These findings indicate that both wavelength and exposure duration influenced the degree of surface alteration, with the 810 nm groups, particularly at 45 seconds, showing the greatest structural and compositional change [8].

The laser interaction with mineralized tissues depends on wavelength, delivered energy, and irradiation time. As the duration of exposure increases, cumulative thermal effects may lead to dehydration of the organic matrix, disruption of mineral content, microcrack formation, and surface melting or charring [7,8]. Aoki et al. emphasized that laser effects in nonsurgical periodontal therapy are highly parameter-dependent and that beneficial debridement must be balanced against the risk of thermal injury to the root surface [10]. The present findings fit well within that concept, since the lower-exposure groups showed relatively limited change, whereas the 810 nm groups demonstrated a marked increase in roughness and more pronounced elemental alteration.

The present results are in close agreement with the in vitro observations of Kreisler et al., who reported that diode laser irradiation can produce morphological alterations on root surfaces depending on the parameters used [7]. A similar trend was reported by Jayachandran et al., who found that increasing 810 nm diode laser exposure caused progressively greater surface irregularity and compositional change, with longer exposure associated with evident charring and carbonization [8]. Taken together, these studies and the present findings suggest that exposure time is a critical determinant of root surface modification when diode lasers are used. The relatively small difference between the two 660 nm groups in the present study further suggests that lower-wavelength irradiation under the tested conditions may remain below the threshold for major surface damage.

The biologic significance of these surface changes should also be considered carefully. Trylovich et al. showed that neodymium-doped yttrium aluminium garnet (Nd:YAG) laser treatment of endotoxin-treated root surfaces resulted in reduced favorable fibroblast attachment compared with control surfaces [11]. Thomas et al. similarly reported that laser-only treatment could adversely affect fibroblast attachment, indicating that laser-induced surface modification does not necessarily improve biocompatibility [12]. In contrast, Kreisler et al. demonstrated that controlled diode laser irradiation may influence periodontal ligament cell attachment in vitro [13], and Choi et al. reported that semiconductor diode laser exposure could promote proliferation and differentiation of human periodontal ligament fibroblasts under specific conditions [14]. These findings suggest that diode laser effects are dose-sensitive. Limited and controlled irradiation may be biologically acceptable, whereas greater surface alteration may reduce the suitability of the root surface for periodontal healing.

Thermal safety is another important consideration. Kreisler et al. demonstrated that intrapulpal temperature rise during root surface irradiation with an 809 nm GaAlAs laser depends strongly on the applied parameters and may approach levels that are potentially harmful to pulp vitality when power and time are increased [15]. Falkenstein et al. also showed that during periodontal treatment with a 940 nm diode laser, heat transmission through the root is influenced by dentin thickness, power setting, and exposure duration, and that some settings may exceed safe thermal limits [16]. The greater roughness and the more pronounced decrease in mineral-related elements in the higher-exposure groups of the present study are therefore consistent with a stronger cumulative thermal burden on the irradiated surface.

From a clinical perspective, these findings support a conservative approach to the use of diode lasers on root surfaces. Systematic reviews have shown that diode lasers may provide adjunctive benefits in periodontal therapy, particularly when combined with RSD, but the benefits are not uniform across all protocols [4,5]. Earlier studies with Nd: YAG lasers also showed that laser irradiation can alter both root surface morphology and subgingival microflora, again highlighting that decontamination and surface preservation must be balanced carefully [17]. In this context, the present findings suggest that lower or shorter exposures may be preferable when the goal is root surface detoxification without excessive structural alteration.

One of the main advantages of the present study is that it evaluated diode laser effects using a controlled in vitro design with equal group allocation and direct comparison of two clinically relevant wavelengths at two exposure durations. In addition, the study combined quantitative surface roughness assessment with SEM-based morphologic evaluation and EDAX-based elemental analysis, allowing both structural and compositional changes to be assessed together. This provides a more comprehensive understanding of root surface response than studies limited to only one outcome measure. The clinical significance of the present findings lies in helping clinicians appreciate that diode laser settings cannot be selected arbitrarily. The results suggest that lower or shorter exposures may better preserve root surface integrity, whereas higher wavelength and longer exposure may be reserved for situations requiring stronger decontamination, provided the risk of surface alteration is taken into account.

The present study has some limitations. It was conducted in vitro on extracted single-rooted teeth, so the findings cannot fully reproduce the effects of blood flow, moisture, periodontal pocket anatomy, tissue hydration, and host healing response seen under clinical conditions. Single-rooted teeth were selected to reduce anatomic variability and permit more standardized laser application and surface analysis. However, this also limits direct extrapolation of the findings to multirooted teeth and furcation-involved surfaces. The study focused on surface roughness, SEM appearance, and elemental profile, but did not assess biologic endpoints such as fibroblast attachment, bacterial reduction, or intrapulpal temperature within the same experimental model. In addition, baseline surface roughness was not recorded before laser application. Although specimens were selected using standardized inclusion criteria, pre-intervention roughness assessment would have allowed a more accurate within-sample comparison and would have further strengthened the study design. Future studies should include untreated control groups, baseline roughness assessment, direct thermal monitoring, specimen-level replicated compositional analysis, and cell-based, animal, or clinical models to identify diode laser settings that provide effective decontamination while preserving root surface biocompatibility and healing potential.

## Conclusions

Within the limitations of this in vitro study, diode laser irradiation produced wavelength- and time-dependent changes in root surface structure and composition. The 810 nm laser, especially at 45 seconds, produced the greatest increase in surface roughness and the most marked alteration in elemental profile, whereas the 660 nm settings resulted in comparatively milder changes. These findings suggest that lower or more controlled diode laser exposure may be preferable when the aim is root surface decontamination without excessive surface alteration. Clinically, the study highlights the importance of careful parameter selection during diode laser use in periodontal procedures, as inappropriate exposure may adversely affect root surface integrity and potentially influence subsequent healing. Further studies under biologic and clinical conditions are needed to identify laser settings that provide therapeutic benefit while preserving root surface compatibility.
